# Measuring human mobility in times of trouble: an investigation of the mobility of European populations during COVID-19 using big data

**DOI:** 10.1007/s11135-023-01678-9

**Published:** 2023-05-13

**Authors:** Barbara Guardabascio, Federico Brogi, Federico Benassi

**Affiliations:** 1 University of Perugia, Perugia, Italy; 2 Italian National Institute of Statistics (ISTAT), Rome, Italy; 3 University of Naples Federico II, Naples, Italy

**Keywords:** Big data, Human mobility, Mobility restriction index, COVID-19, Exogenous shocks, Fast demography

## Abstract

Spatial mobility is a distinctive feature of human history and has important repercussions in many aspects of societies. Spatial mobility has always been a subject of interest in many disciplines, even if only mobility observable from traditional sources, namely migration (internal and international) and more recently commuting, is generally studied. However, it is the other forms of mobility, that is, the temporary forms of mobility, that most interest today’s societies and, thanks to new data sources, can now be observed and measured. This contribution provides an empirical and data-driven reflection on human mobility during the COVID pandemic crisis. The paper has two main aims: (a) to develop a new index for measuring the attrition in mobility due to the restrictions adopted by governments in order to contain the spread of COVID-19. The robustness of the proposed index is checked by comparing it with the Oxford Stringency Index. The second goal is (b) to test if and how digital footprints (Google data in our case) can be used to measure human mobility. The study considers Italy and all the other European countries. The results show, on the one hand, that the Mobility Restriction Index (MRI) works quite well and, on the other, the sensitivity, in the short term, of human mobility to exogenous shocks and intervention policies; however, the results also show an inner tendency, in the middle term, to return to previous behaviours.

## Introduction

Spatial mobility is a distinctive feature of human history (Bacci 2017, 2018). Many efforts have been made by scientists from different disciplines, from geography to physical statistics, to understand its mechanisms and thus generalise them in the form of a law. The pioneering contributions of Ravenstein (1885, 1889) have since been joined by many other attempts, most notably the contributions of Schläpfer et al. (2021), Alis et al. (2021). However, for a long time, human mobility was studied and approached by considering migration, a type of mobility that is less temporary than non-residential mobility and results in variations in the sizes and structures of the departure and arrival populations (Skeldon 2017). There are several reasons for this increased attention. First, there is a question of data availability; classic migration (typically labour migration) involves flows of people that are widely recorded by official statistics and for which there has always been relatively good data availability. Moreover, these types of migrations, which have characterised the industrial development of the Western world and in particular that of Europe, according to the rural–urban migration pattern, are less complex forms of mobility to measure and thus to interpret and model. Of all the models, it is enough to recall here the famous model of Harris and Todaro (1970), which formalised the relations between push and pull factors in labour migration, and the more general multi-exponential model of Rogers and Castro (1981), which laid the foundations for modelling, in a predictive sense, migration and age structures. In reality, however, as has been well known for many years now, the form of spatial mobility that most affects human activities is not the permanent form but rather that of temporary movements (Bottai and Barsotti 1994). This form of mobility has grown a great deal in recent years and is the form of mobility that, to date, has been observed the least and only partially. There are many reasons for this growth. As theorised by Zelinsky in his seminal contribution on the mobility transition half a century ago (Zelinsky 1971), this form of mobility (i.e. temporary, and increasingly fast and frequent) is characteristic of hyper-advanced modern societies. In these types of societies, in fact, people tend to stay close to each other in space, typically in urban and hyper-urban contexts—it is no coincidence that as of 2007, the world’s population is urban for the first time in history—where there are many interconnections and the infrastructures for both land and area mobility exist. This increase in mobility (in terms of both demand and supply) has been matched, at least until now, by a tendency to decrease the (monetary) costs associated with it. This general pattern has meant that in a relatively small number of years, i.e. during the period of the globalisation of economies, we have witnessed a process of the compacting and shrinking of space that has, in fact, globalised not only the exchange of capital and goods but also of people by drastically increasing spatial mobility on both macro- and micro-scales and changing the relative proportions of its forms (Tjaden 2021). Space has become so narrow and compact that it is no longer easy to understand what is near and what is far and thus to distinguish between long-haul and short-haul mobility. As to the reasons for the lack of observations, a predominant role has been played by data (statistical information), which until now, with respect to these forms of migration, were very few and only related to specific types of non-residential mobility, such as commuting movements recorded by population censuses. The situation has, however, changed rapidly, and this is also thanks to the technological revolution that has made it possible to obtain huge amounts of information on human mobility from new data sources and, specifically, from big data and digital data. This availability of data therefore makes it possible to measure and model these forms of mobility, which, being more elastic than traditional ones, are hyper-sensitive to sudden exogenous shocks such as extreme natural events and economic and health crises. The field of measuring these forms of mobility is quite new and therefore there are many challenges, including methodological ones, that need to be addressed. The implications, however, can be enormous because having reliable methodologies for measuring these forms of mobility makes it possible to prepare real-time-based policies that can make a difference in terms of spatial planning and measuring the effectiveness of certain political actions. Based on these premises and in line with some recent studies on mobility during the COVID-19 pandemic (Li et al. 2021; Lucchini et al. 2021; Hu et al. 2021), this contribution intends to provide empirical evidence on the measurement of the mobility of the European population during COVID-19. In particular, by exploiting Google mobility data, a Mobility Restriction Index (MRI) is formalised based on the different mobility restrictions imposed by different governments due to COVID-19, and it is also able to take into account the behaviours adopted by different citizens with respect to these restrictions. The index, which is sensitive to both the effects of COVID-19 on mobility and the effects exerted by the refugee flows caused by the Russian-Ukrainian War, is based on daily data and thus is theoretically sensitive to mobility restrictions. This is the main difference between this index and the better-known Stringency Index (SI) proposed by Oxford University (Ritchie et al. 2020). The MRI is calculated for Italy for the period from February 2020 to April 2022, as well as for the other 26 EU countries. It is then compared with the SI for both comparative and interpretative purposes. It is important to clearly explain that the aim of the index and therefore of the paper itself is to detect the reduction in human mobility caused by the restrictions adopted by governments in order to limit the spread of the COVID-19 virus. Thus, to a certain extent, we measured the resistance to movement. This implies that our index had low values when people did not comply with the constraints imposed by their governments and measured more accurately by the Oxford Stringency Index. The rest of the paper is structured as follows: Sect. 2 provides a review of the literature that focuses on the transition from migration to digital mobility. Section 3 presents the Google mobility data and introduces the MRI methodology, and Sect. 4 applies the suggested methodology in order to measure the mobility structure in Italy and provide a comparison of the COVID-19 outbreak responses imposed by each European government. Finally, Sect. 5 contains some comments and conclusions.

## Migration, mobility, and digital demography. Theories, old constraints, and new empirical possibilities

Historically, scholars have long attempted to find the causes and functional mechanism of migration. One of the earliest attempts that had a strong impact on the literature can be traced back to the work of the German geographer Ravenstein, who formulated the so-called ‘migration laws’ (Ravenstein 1876, 1885, 1889).

Ravenstein’s seminal work and his many insights into functional mechanisms that can explain and, to some extent, predict migration (like, for example, the gravitational approach) have significantly influenced theoretical approaches and interpretative models on internal and international migration. In this regard, one can consider the works of Lee (1966), Tobler (1995), Simini et al. (2012), Piché and Dutreuilh (2013) and Stillwell and Thomas (2016).

Based on Ravenstein’s migration laws, theories and interpretative approaches have proliferated over time (King 2012). Despite the strong expansion of new interpretative paradigms, including the idea of living in the so-called ‘age of migration’ (Castles et al. 2014), the study of migration still does not have its own migration theory. Rather, scholars basically agree that there is a fairly heterogeneous set of theories that are ‘valid’—that is, verifiable—for particular geographical contexts and at defined historical moments. In short, migration theory is very rich (for an exhaustive review, see Massey et al. (1993)), but at the same time it is weak (Arango 2017; Carling et al. 2020).

The interpretative and explanatory limits of migration theories depend, among other things, on the complex and mutable nature of the concept of ‘migration’ itself. Migration (in its traditional form) implies a non-temporary movement that, on the one hand, presupposes a migratory project of the migrant subject and, on the other, causes changes in the sizes and structures of the origin and destination populations. In fact, migration causes a decrease in the size of the population of the place of origin and, automatically, causes an increase in the size of the destination population. Since those who migrate usually have a young average-age structure, one of the effects of migration is also the transfer of potential new births and a rejuvenation in the age structure of the populations of the destination countries (Plane 1993; Rogers et al. 2002). However, ‘classical’ migration (often called labour migration) is only one form of mobility, and, moreover, it is progressively less relevant (in numerical terms) in the contemporary world, where spaces are highly compacted (Miller 2004) and the underlying causes of migration flows are no longer only economic (think, for example, of migration induced by climate change). Even with respect to mobility, however, interpretative schemes, theories, and of course modalities have changed a great deal in the recent past. According to classical theory, everyday mobility (typically labour mobility) was a form of systematic spatial interaction closely connected with migration and linked to the overall cost function (home and commuting costs) determined by the worker’s need to cover the distance between home and work given a certain wage Baccaïni (1997) and Bottai and Barsotti (2006). However, as can be guessed, many years ago this form of everyday mobility was only a part (albeit a significant one) of general mobility (which clearly included all, and even non-systematic, trips, such as those made for shopping, parental care, cultural consumption, etc.). The study of these forms of mobility, or the use of space, was of course hypothesised but almost never carried out, given that the only source of data for measuring the daily mobility was the population census, which, as is well known, historically surveyed mobility for work purposes and only more recently has been used to collect data for study purposes. Apart from scattered cases of ad hoc sample research (Bottai and Benassi 2016), all other forms of mobility were undetected, even though they existed and affected the lives of individuals and the definition of living spaces.

The distinction between migration and (non-definitive) mobility has been increasing over time; this is also an effect of the social transformations induced by technological diffusion and the compaction of space. Migration obviously remains a form of mobility, but there are wide and growing differences between the two that do not just concern the temporariness of spatial movement. Another fundamental difference is the speed at which the movement is realised. If it is true that migration, compared to other demographic events such as births and deaths, can be considered a ‘fast’ event Billari (2022), it is equally true that compared to mobility, it is still a slow process. We refer in particular to sudden (i.e. non-systematic) and very random forms of mobility such as, for example, those induced by sudden exogenous shocks (war, environmental disasters, pandemic crises, etc.). However, it is also true that the areas of overlap between the different forms of mobility are wide, and to some extent, the distinctions between one form of mobility and another are often very blurred. This is also because contemporary migration does not generally imply a lasting project and a definitive change; one thinks, for instance, of all those forms of migration linked to de facto temporary movements for work, education, adventure, etc.

From this perspective, the possibilities offered by the digital world and the nascent science of digital and computational demography are manifold, and in recent years, there have been many attempts to quantify human mobility (and related aspects) using digital data (from Google, Facebook, LinkedIn, etc.). Particular attention has been paid to the quantification of migrant stocks and, in recent times, to the quantification of mobility as a response to sudden and impactful exogenous shocks, especially because of the considerable implications these processes have for the preparation of intervention plans and policy programming.

The first strand includes the works of Napierała et al. (2022) and Rowe (2021) on using the Facebook platform to monitor migrant stocks, the contribution of Rampazzo et al. (2021) on defining a framework for estimating migrant stocks by combining digital footprints and survey data, and also the seminal contribution of

Alexander et al. (2022) on the combined use of social media and survey data to nowcast migrant stocks in the United States. Regarding mobility due to exogenous shocks and its impacts at the territorial level, it is worth mentioning the work of Alexander et al. (2019) on using Facebook data to determine the impact of Hurricane Maria on emigration from Puerto Rico. More recent contributions analysed the effects of COVID-19 on the temporary population of Rome using Facebook data (Brollo and Celata 2022) and the link between mobility and mortality in England and Wales using Google mobility data (Basellini et al. 2021). Finally, Rowe et al. (2023) analysed human mobility in Britain during the COVID-19 pandemic using meta-Facebook data.

All these contributions have shown that, even with some limitations related to the nature of the data sources used, indicators and measures built on digital data can be used as an early warning system for changes in migration and mobility (Barker and Bijak 2022; Napierała et al. 2022).

Our contribution fits into this strand of studies; we investigate a particular aspect of mobility or, rather, resistance to mobility. On the basis of data obtained from Google and referring to the daily mobility of individuals in selected European countries, we quantified the degree to which individuals adhered to the restrictions on mobility imposed by different governments to curb the spread of COVID-19. The idea is to understand, using an ad hoc constructed index and on the basis of the Google data, where and to what extent the mobility of individuals adapted to the restrictions imposed and, thus, to evaluate, in essence, the effectiveness of different governments’ policies in restricting the mobility of individuals. This is an entirely original theme compared to the most recent studies that used digital sources to study migration and mobility processes.

## Data and method

### The Google mobility data

The Google COVID-19 Mobility Report data are available from the start of the pandemic to the present; these data include 6 daily time series whose time span is 15 February 2020 to today. These time series provide the daily variations that show how visits to several types of places have changed in each geographic region since February 2020 due to the pandemic. Mobility trends are defined for the following categories of places, which include places that are useful for social distancing efforts and places that provide access to essential services:

- Grocery & pharmacy (grocery stores, food warehouses, farmers’ markets, specialty food shops, drug stores, and pharmacies);
- Parks (local parks, national parks, public beaches, marinas, dog parks, plazas, and public gardens);
- Transit stations (public transport hubs, such as subway, bus, and train stations);
- Retail & recreation (restaurants, cafes, shopping centres, theme parks, museums, libraries, and movie theaters);
- Residential (places of residence);
- Workplaces (places of work).

In more detail, these datasets show how visits to and the length of time spent at different places change compared to a baseline. The baseline value for each day of the week is set as the median value for the corresponding day of the week during the 5-week period from 3 January to 6 February 2020. The datasets show trends over several months.^1^

### Measuring mobility restriction

Using the Google mobility data, we perform a principal component analysis (PCA) in order to construct an indicator that is able to explain the level of restriction imposed by a given country. Based on the estimation of the eigenvalues of the sample covariance matrix, the classical PCA developed by Pearson (1901) and Hotelling (1933) is an unsupervised learning approach that is meant to reduce the dimensionality of multivariate data while preserving as much of the relevant information as possible. Over the years, PCA has been employed in the literature to perform the following tasks:

- to improve the macroeconomic forecasting performance by reducing the data dimension when a large set of predictors is available (see, inter alia, Forni et al. (2005) and Stock and Watson (2002));
- to create a sentiment measure representing the optimistic or pessimistic views of investors (Baker and Wurgler 2006);
- to create a policy uncertainty index using newspaper coverage of policy-related economic uncertainty (Baker et al. 2016).

Factor analysis is a related problem that is distinct from PCA, just as it is in a low-frequency setting (see, among others, Bai and Ng 2002; Stock and Watson 2002; Kapetanios 2010; Cubadda and Guardabascio 2012). The above factor models are static, as opposed to the dynamic factors discussed in Forni et al. (2000) and their extensions; frequency-domain PCA is applied for these dynamic factors. More recently, Wu and Zaffaroni (2018) provided uniform convergence results of lag-window spectral density estimates for a general class of multivariate stationary processes, while Aït-Sahalia and Xiu (2019) found consistency between the low- and high-frequency PCA structures and showed that in the presence of high-frequency data, the first component becomes increasingly dominant. Let us suppose that $X_t$ is an *N*-vector of high-frequency time series whose covariance matrix is given by $E(X_t X'_t) = \varSigma _{xx} = \varPsi \varLambda \varPsi$, where $\varPsi$ is the eigenvector matrix (loadings) of $\varSigma _{xx}$ and $\varLambda$ is the associated diagonal eigenvalue matrix (scores). It is possible to estimate our restrictive mobility index by simply keeping the first principal component, which is equal to

1 $$\begin{aligned} F_t = \varLambda _1 \varPsi '_1 X_t. \end{aligned}$$

Once the first principal component $(F_t)$, which includes the largest part of the mobility variability, has been extracted, in order to obtain an index whose values are between zero and one, we rescale it using the following formula:

2 $$\begin{aligned} z_t = \frac{F_t-min(F_t)}{max(F_t)-min(F_t)}. \end{aligned}$$

We obtain a new indicator $z_t \in [0,1]$; a value equal to 1 indicates that the selected country has imposed a total lockdown, and the index will be close to zero in the opposite case. The new indicator will explain the changes in mobility flows due, for example, to COVID restrictions imposed by each government during the pandemic, or it can be used to map refugees flows during the Ukraine War.

## Results

### The mobility restriction index: the case of Italy

Starting in February 2020, many governments put in place a whole range of containment and closure policies to attempt to contain the spread of COVID-19, including closing schools and businesses and sometimes even preventing people from leaving their homes except for essential reasons.

These policies varied considerably across governments, in terms of both the level of stringency and the length of time that authorities allowed them to stay in place. The main effect of these policies was reducing mobility, so that they appear to be in conflict with individual freedom.

The Italian government was the first government in Europe to declare (on 31 January 2020) a state of emergency that continued until April 2022; the Italian government was also the first European government to impose severe rules restricting mobility. More recently, in the opposite direction, the Russia-Ukraine War has led to an increase in mobility due to the influx of thousand of refugees.

Table 1 summarises the main restrictions imposed by the Italian government from February 2020 to April 2022; the restrictions responsible for the most significant changes in mobility are highlighted in gray.

**Table 1:** Main mobility measures adopted by the Italian government from February 2020 to April 2022

N.	Date	Measure	Main step
1	23/02/2020	Red zone creation	COVID-19 Pandemic—Phase 1
2	25/02/2020	Urgent measures to contain spread	
3	01/03/2020	Further measures to protect areas at risk	
4	08/03/2020	Two levels of restrictions	
5	09/03/2020	Red zone extended to whole country	Lockdown
6	11/03/2020	Lockdown	
7	22/03/2020	Closure of all non-essential commercial activities	
8	28/03/2020	Rules for travellers entering Italy	
9	01/04/2020	Extension of all previous restrictions to 13 April	
10	10/04/2020	Extension of all previous restrictions to 3 May	
11	04/05/2020	End of lockdown	End of Lockdown—Phase 2
12	15/06/2020	Decrease in containment measures	
13	08/10/2020	New containment measures	New Containment Measures—Phase 3
14	06/11/2020	Curfew and establishment of yellow, orange, and red zones	
15	21/12/2020	Lockdown	Lockdown
16	16/01/2021	Establishment of white zones	End of lockdown
17	06/03/2021	Reinforcement of containment measures	Reinforcement of containment measures
18	26/04/2021	Decrease of containment zone and Green Pass	
19	06/08/2021	Green Pass obligation	Green Pass Obligation—Phase 4
20	06/12/2021	New restrictions and vaccination Green Pass	Vaccination Green Pass
21	24/02/2022	Russia-Ukraine War	Russia-Ukraine War
22	01/04/2022	End of COVID-19 state of emergency	End of COVID-19 state of emergency

The most significant events are highlighted in gray

**Fig. 1:**
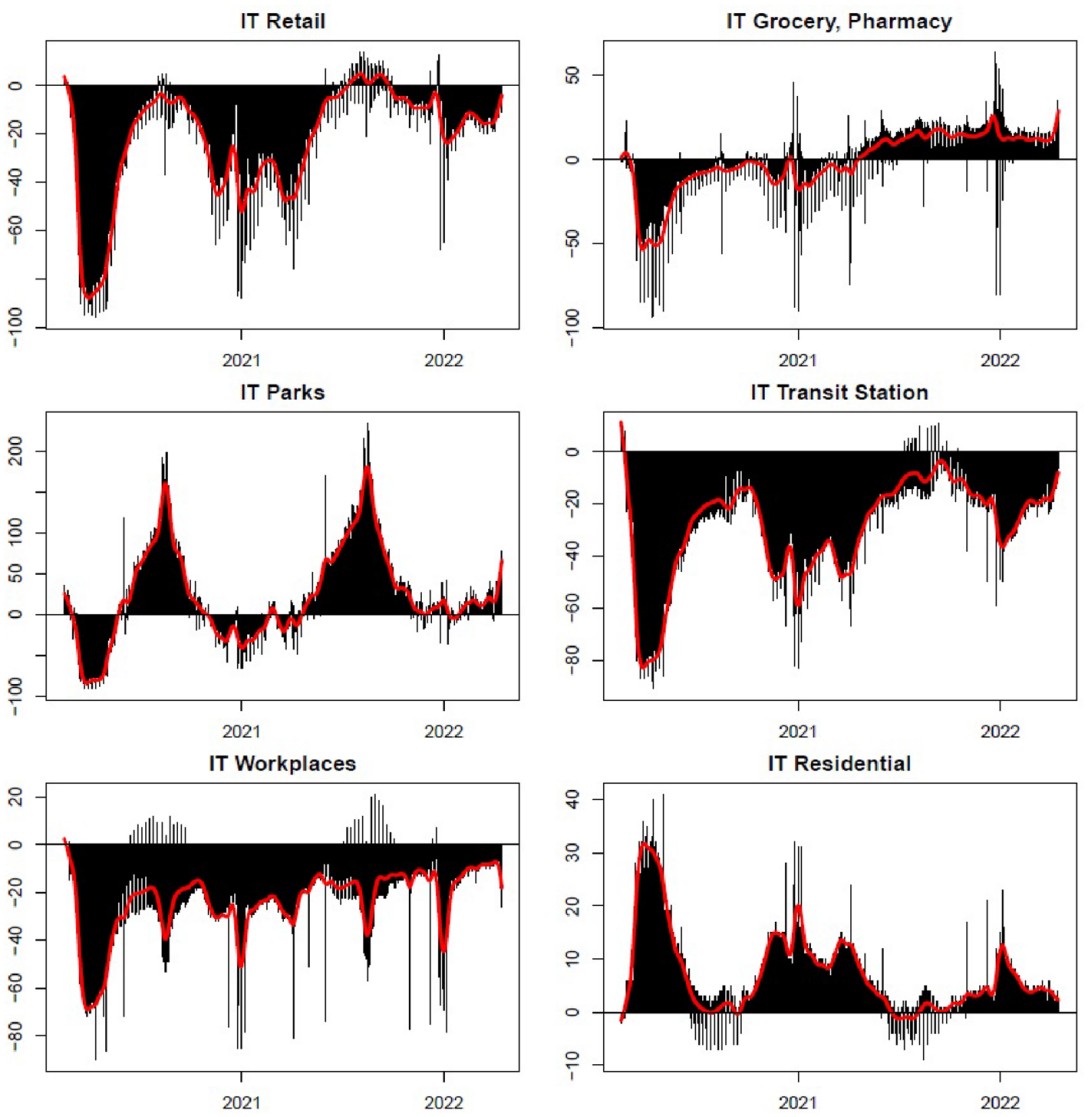
Daily Google mobility data for Italy from February 2020 to April 2022

The results indicate that, from the first daily release of Google mobility data to today, the pandemic and then the Ukraine War caused the mobility inside each country and among nations to change drastically. In more detail, the policies adopted by the Italian government caused travel to shops, parks, workplaces, and transit stations to largely decrease in favour of residential places, while the grocery and pharmacy category follows its own path, as shown in Fig. 1.

**Fig. 2:**
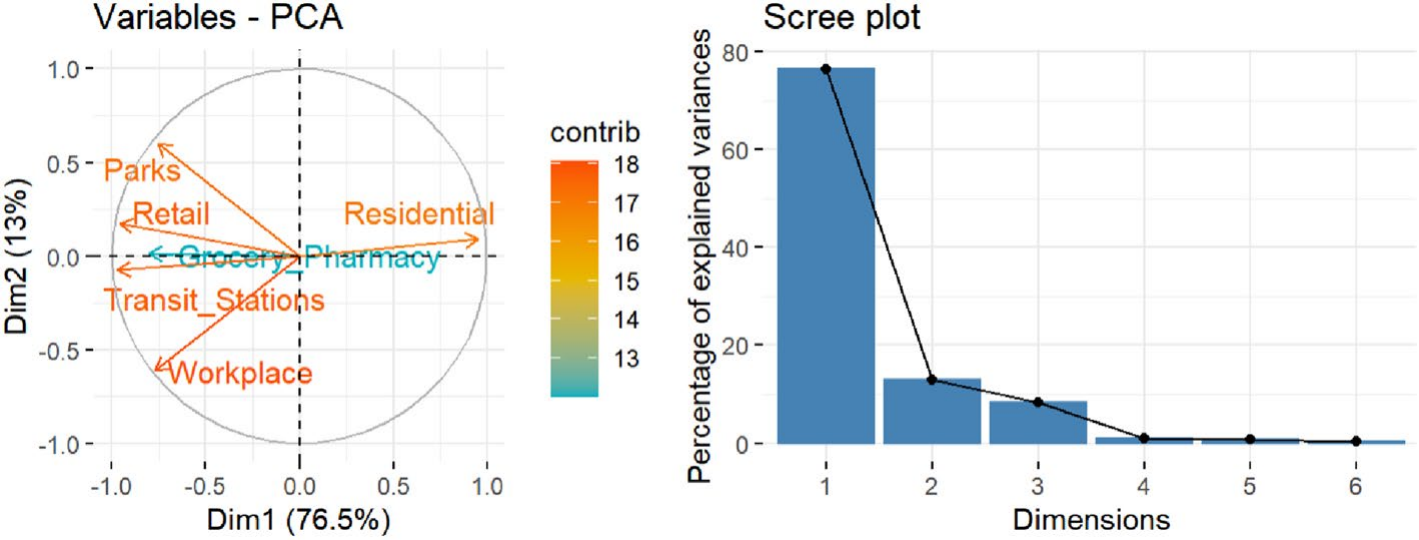
Principal component analysis results: Loadings and percentage of the variance explained by each dimension

Given this set of data, we apply principal component analysis, as described in Sect. 3.2, to obtain the Mobility Restriction Index; the main results are displayed in Fig. 2, which includes the following:

- A scree plot (on the right side) displaying how much variation each principal component captures from the data.
- A biplot (on the left side) representing the variable vectors starting from the origin ([0,0]) to the coordinates given by the loading vector. The direction of each vector defines its contribution to a PC: the more parallel a vector is to a principal component axis, the more it contributes only to that PC. The length in space of each vector defines the amount of variability of the corresponding variable that is represented by the displayed PCs: larger vectors are better represented by the PC being considered. The loading plots also hint at how the variables are correlated with one another: a small angle implies a positive correlation, a large angle suggests a negative correlation, and a 90$^\circ$ angle indicates that there is no correlation between two features.

In this case, in line with Aït-Sahalia and Xiu (2019), the results suggest that the first component represents the most variation (76.5%) of the total dataset. This component is positively correlated with the residential category and negatively correlated with all the other variables. All the variables are largely represented, but the grocery and pharmacy category has the lowest contribution, although it is fully correlated with the first component and totally excluded by the second one.

**Fig. 3:**
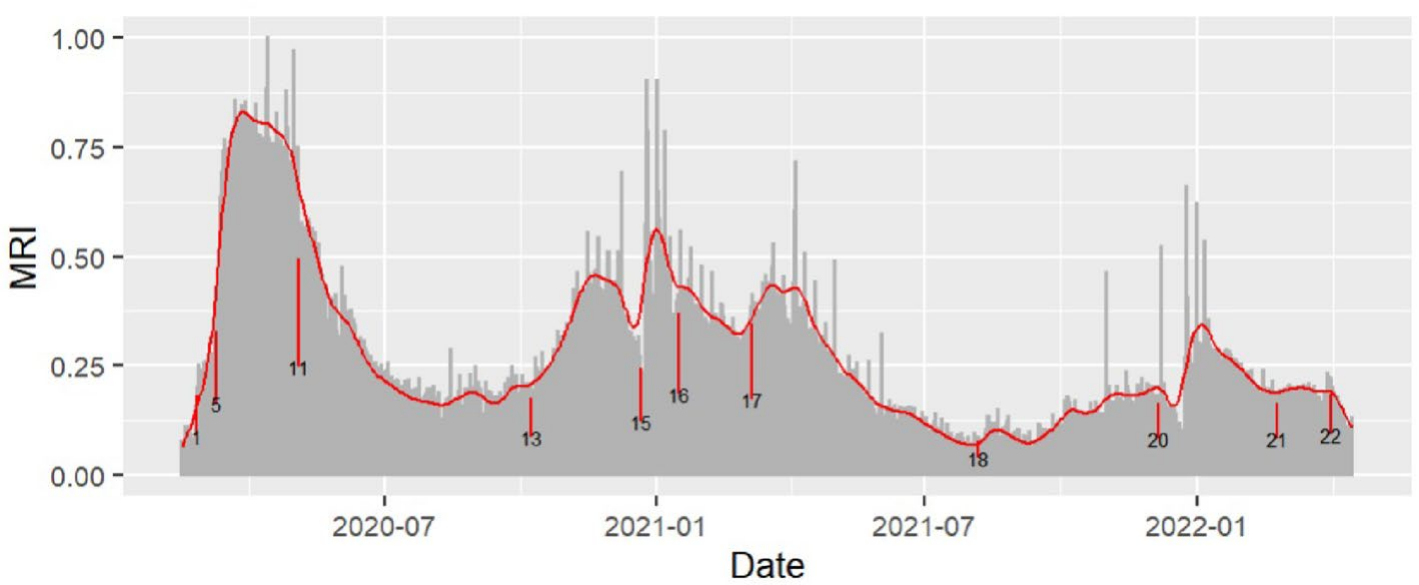
Time series of the MRI for Italy and major events from February 2020 to April 2022

Now that we have proved that the first principal component is fully representative of the greatest part of the information included in our dataset by applying the transformation in Eq. 2, we can finally obtain our Mobility Restriction Index. An important task in the exploration of time series data is analysing the ability of the data to detect and react to significant events that are highly correlated with the phenomenon represented. In Fig. 3, we display the estimated policy indicator for Italy and the events connected to the main measures, which were highlighted in gray in Table 1.

Looking at these results, we can see that our MRI precisely follows the path of the mobility restrictions imposed by the government. Indeed, from Fig. 3, we can see that on the left side, the lockdown period (between March and April 2020—from point 5 to point 11) is well represented, followed by a decrease in the restriction level during the summer (point 11 to point 13) and the new lockdown introduced at the end of 2020 (points 15 and 16); finally, we have a new increase due to the reinforcement of containment measures in March 2021 (point 17) and then another decrease after the introduction of the Green Pass. The last part of the series features the vaccination Green Pass restriction (point 20), which ended in March 2022 and whose effect was partially reduced by the flows of refugees from Ukraine (i.e. the indicator is decreasing because people began to move due to the effects of the Ukraine War).

To assess the effectiveness of our indicator, we compare itwith the Oxford Stringency Index, which measures government responses to the COVID-19 outbreak. Created to track and compare the responses of governments around the world, the Oxford Stringency Index is an aggregate score / composite measure made up of a particular combination of policy indicators / response metrics. It includes nine main categories: *school closures, workplace closures, cancellations of public events, restrictions on gatherings, public transportation closures, stay-at-home requirements, restrictions on internal movement, restrictions on international travelling, and public information campaigns*. This index ranges from 0–100 and is based on the policies and statements of each government, and for these reasons, it appears to be flat in the absence of new interventions.

**Fig. 4:**
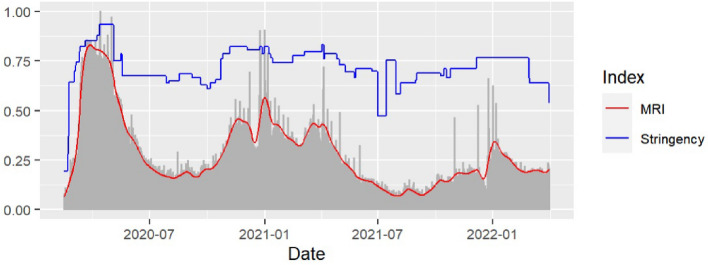
Time series of the MRI and Stringency Index for Italy from February 2020 to April 2022

Our MRI indicator analyses the effect that restrictions have on the population in terms of reduced mobility and also incorporates compliance behaviours; consequently, it presents a larger variability (Fig. 4).

By comparing the paths of the two indices, we can see that they present quite similar dynamics during the first lockdown, when few people left their own homes. After 3 May 2020, the two indices start diverging.

This result can be explained by the individual responses of the population to the restrictions: people had probably gotten used to living with the risk of the pandemic, and they did not comply with rules and voluntary restrictions on mobility and social interactions as consistently.

Moreover, during the second wave, the restriction system was introduced on a regional basis; governments’ restrictions were more localised and/or targeted at specific activities compared with the first wave, and the tiers of these restrictions became dependent on the combination of 21 quantitative indicators.

Due to the complexity of the regulatory framework, any comparison of the restrictions adopted during the first and second waves appears to be difficult and even, in some cases, inconsistent. In particular, in Italy, the new regional system caused the restriction level defined by the Oxford Stringency Index for the second wave to be overestimated (Conteduca 2021).

This result is also highlighted by Table 2, which shows some summary statistics of the two indexes. In particular, over the period considered, the Stringency Index presents a larger mean and a smaller variability compared to the MRI.

**Table 2:** Descriptive statistics for the mobility restriction index and stringency index in Italy

Index	MRI	Stringency
Min	0.0664	0.1944
1st Quartile	0.1665	0.6759
Median	0.2123	0.7130
Mean	0.2862	0.7225
3rd Quartile	0.3808	0.7778
Max	0.8305	0.9352
Variance	0.0324	0.0088
IQ Range	0.1685	0.1019

Moreover, although the two indices appear to be correlated, if we consider the full timespan (15 February 2020 to 30 April 2022), we obtain a correlation equal to 0.68. The correlation value changes substantially if, following the scheme shown in Table 1, we split the full sample into four subsamples:

1. *Phase 1 (31 January 2020 to 3 May 2020)*: The first COVID wave featured more generalised lockdown measures. In this phase, the correlation between the two indices is equal to 0.85.
2. *Phase 2 (4 May 2020 to 7 October 2020)*: Once the first COVID wave had ended, containment measures slowly started to decrease. In this period, the MRI is largely smaller than the Stringency Index, and the correlation between the two is equal to 71%.
3. *Phase 3 (8 October 2020 to 5 August 2021)*: The second COVID wave caused new restrictions to be introduced but, unlike the restrictions from the first COVID wave, they were applied on a regional basis. This tendency toward the localisation of health measures caused the national Stringency Index to overestimate the level of restriction. For this reason, during this period, the correlation is stable at 71%.
4. *Phase 4 (6 August 2021 to the present)*: The second wave had ended, and the Green Pass had been introduced in Italy. During this period, the Russia-Ukraine War had an impact on mobility that was the inverse of the impact of the COVID restrictions; as a result, the correlation between the two indexes decreases to 59%.

### MRI for European countries

Now that we have analysed the main features of our MRI and compared it with the Stringency Index, we can look at the results obtained for all the European countries.

**Fig. 5:**
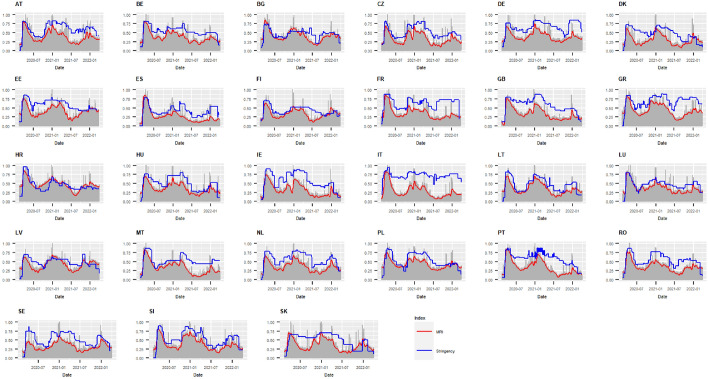
Time series of the MRI and Stringency Index for the other EU countries from February 2020 to April 2022

For this purpose, in Fig. 5, we display a comparison of the MRI and Stringency Index for all EU countries. For some of these countries, such as France and Greece, we notice a behaviour similar to that of Italy; indeed, for these countries, after the first COVID wave, the two indicators appear to be more distant from each other. However, for some of the other countries, we can see that the two indicators overlap for a longer period of time.

To deepen this analysis, we compare some summary statistics for the two indicators for various countries. In Table 3, we present the mean, median, variance, and interquartile range for both the MRI and the Stringency Index, together with their correlation, for each EU country.

**Table 3:** Descriptive statistics of the MRI and stringency index for all EU countries

Country	MRI				Stringency				Correlation
	Mean	Median	Variance	IQR	Mean	Median	Variance	IQR	
AT	0.39	0.36	0.02	0.22	0.60	0.60	0.03	0.26	0.75
BE	0.38	0.34	0.02	0.19	0.54	0.51	0.02	0.16	0.78
BG	0.41	0.39	0.02	0.18	0.46	0.48	0.02	0.15	0.68
CZ	0.33	0.29	0.02	0.28	0.52	0.48	0.03	0.23	0.85
DE	0.40	0.36	0.01	0.16	0.66	0.68	0.02	0.21	0.59
DK	0.29	0.25	0.02	0.20	0.47	0.51	0.03	0.23	0.51
EE	0.41	0.40	0.02	0.19	0.58	0.61	0.02	0.23	0.54
ES	0.26	0.23	0.03	0.16	0.41	0.38	0.02	0.22	0.69
FI	0.33	0.32	0.02	0.20	0.43	0.41	0.01	0.18	0.60
FR	0.35	0.30	0.02	0.19	0.63	0.68	0.02	0.23	0.56
GB	0.33	0.29	0.03	0.25	0.58	0.62	0.04	0.25	0.80
GR	0.43	0.39	0.03	0.26	0.69	0.74	0.03	0.19	0.45
HR	0.47	0.46	0.02	0.14	0.45	0.41	0.03	0.18	0.73
HU	0.36	0.32	0.02	0.21	0.50	0.52	0.04	0.40	0.80
IE	0.31	0.26	0.03	0.26	0.58	0.53	0.06	0.37	0.81
IT	0.29	0.21	0.03	0.21	0.72	0.71	0.01	0.10	0.69
LT	0.35	0.30	0.02	0.23	0.46	0.46	0.04	0.32	0.82
LU	0.37	0.34	0.02	0.17	0.47	0.47	0.02	0.14	0.77
LV	0.42	0.43	0.02	0.24	0.48	0.47	0.02	0.15	0.51
MT	0.33	0.32	0.03	0.24	0.52	0.53	0.02	0.09	0.70
NL	0.36	0.34	0.02	0.20	0.56	0.58	0.03	0.30	0.80
PL	0.43	0.40	0.01	0.18	0.52	0.45	0.04	0.32	0.74
PT	0.34	0.28	0.04	0.29	0.57	0.61	0.04	0.25	0.70
RO	0.36	0.34	0.02	0.14	0.55	0.54	0.03	0.21	0.75
SE	0.33	0.30	0.01	0.19	0.52	0.48	0.03	0.32	0.71
SI	0.39	0.33	0.03	0.21	0.53	0.50	0.04	0.34	0.85
SK	0.35	0.31	0.02	0.27	0.49	0.56	0.03	0.28	0.47

The results suggest that for all countries except for Croatia (HR), the Stringency Index is greater than the MRI on average. In more detail, the mean distance between the two indicators is equal to 0.17, with a maximum absolute value of 0.43 for Italy and a minimum of 0.02 for Croatia, which is also the only country that presents a negative sign for this value.

Considering the full timespan (31 January 2020–30 April 2022), the mean level of mobility restriction is equal to 0.36, which is associated with a mean stringency level of 0.53. The lowest level of mobility restriction is associated with Spain, while the highest level of mobility restriction is associated with Croatia. On the other hand, if we look at the value of the Stringency Index, the highest value is associated with Italy (0.72), and the lowest value is associated with Spain.

The two indicators have a correlation equal to 0.69 on average; the maximum value of the correlation is associated with Slovenia (0.85), and its minimum value is associated with Greece (0.45).

**Fig. 6:**
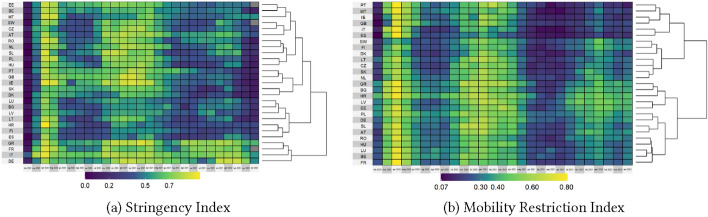
Heatmap and clustering of the Stringency Index and MRI for the EU countries from February 2020 to April 2022

To continue the comparison among countries, we construct two heatmaps based on the averages of the two monthly indicators. As we can see, while the scale of the Stringency Index goes from 0 to 1, the range of the Mobility Restriction Index is between 0.07 and 0.8.

According to the Stringency Index values, Fig. 6 shows four main groups of countries:

- The highest level of restriction for the full sample being considered is adopted by Germany, Italy, France, and Greece.
- The wave in March and April 2020 is the only wave in which there was a lockdown for Spain, Finland, Croatia, Lithuania, Latvia, Bulgaria, and Luxembourg. At later times, their Stringency Index values are almost always lower than 0.5.
- All the other countries (Denmark, Slovakia, Ireland, the United Kingdom, Portugal, Hungary, Poland, Slovenia, the Netherlands, Romania, Austria, the Czech Republic, Sweden, Malta, Belgium, and Estonia) present two main windows of restriction (March and April 2020 and August 2020 to May 2021); some differences among them in the level of restriction imposed can be noticed starting in June 2021.

From the MRI map, we can clearly see the restrictions due to the two COVID-19 waves from March 2020 to May 2020 and from November 2020 to April 2021 during which there were generalised mobility restrictions for all the EU countries. Meanwhile, besides these waves, the following trends in mobility occur:

- Portugal, Malta, Ireland, the United Kingdom, Italy, and Spain feature more mobility.
- Greece, Bulgaria, Croatia, Latvia, and Estonia appear to present a lower level of mobility.
- Germany, Slovakia, Austria, Romania, Hungary, Luxembourg, Belgium, and France, together with Finland, Denmark, Latvia, the Czech Republic, Slovakia, and the Netherlands, besidesbe the two main COVID windows, alternate between periods of greater mobility restrictions and periods of greater freedom.

Finally, we create a heatmap of the difference between the two indicators (Stringency Index—MRI). Interestingly, Italy is the only country with a difference level that is always equal to or greater than zero. This result is in line with Conteduca (2021), proving that after the end of the first wave in May 2020, the Stringency Index overestimates the level of restriction for Italy, extending the regional level of restriction to the whole national territory.

**Fig. 7:**
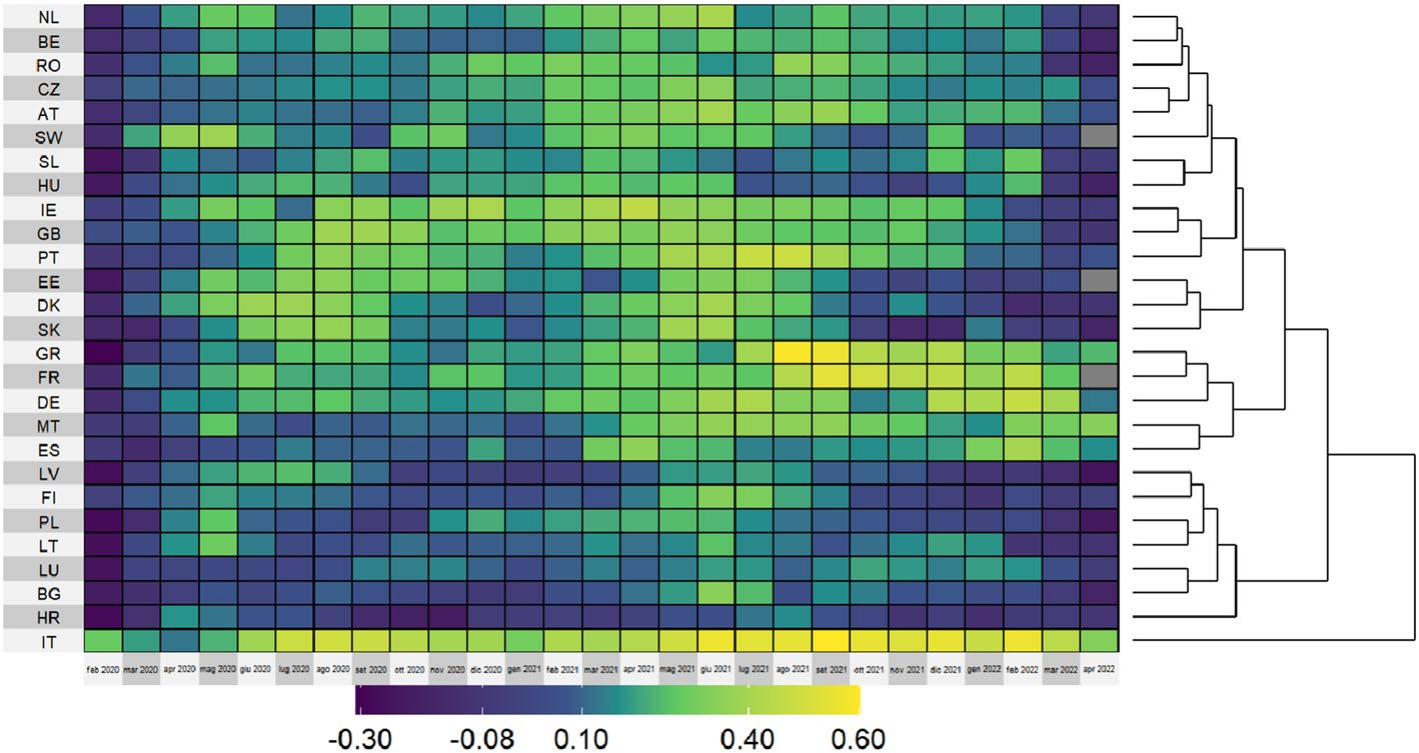
Heatmap and clustering of the difference between the Stringency Index and MRI for EU countries from February 2020 to April 2022

We can categorise the other countries into three main groups:

- The first group is composed of Latvia, Finland, Poland, Lithuania, Bulgaria, and Croatia; for these countries, the two indicators present an overlapped structure, so that the difference between them is almost always around zero.
- The second group includes Greece, France, Germany, Malta, and Spain; for these countries, the two indicators have similar paths until March 2021, and then the Stringency Index becomes higher than the MRI.
- The third group contains all the other countries: Slovakia, Denmark, Estonia, Portugal, the United Kingdom, Ireland, Hungary, Slovenia, Sweden, Austria, the Czech Republic, Romania, Belgium, and the Netherlands have lower Stringency Index values in the two tails (February 2020 to April 2020 and November 2021 to April 2022). In the middle of the full timespan, the scenario changes a little, and depending on the distance between the two indicators, we can define three other clusters, although they are less distant from each other (for more details, see Fig. 7).

## Discussion and conclusions

The importance of using non-traditional sources (and approaches) to study human population changes due to mortality, fertility, migration, and mobility has been recently underlined by a book published by the European Union on ‘Data innovation in demography, migration and human mobility’ (Bosco et al. 2022). New opportunities arising from innovative data sources also pose new challenges related to new measurement tools and analysis methods. It is these challenges that must be met in order to be able to provide governments with timely and accurate data on which to base territorially targeted and thus more effective policies. In this general context, mobility studies play a key role. Indeed, mobility is a phenomenon pertaining to what is known as fast demography (Billari 2022). In particular, as we have been able to verify empirically with this study, temporary mobility, i.e. mobility that does not necessarily result in a change of residence, is extremely sensitive to exogenous factors such as, for example, pandemic crises and the consequent mobility intervention policies. The results showed that the MRI precisely follows the path of the mobility restrictions imposed by governments. However, this trend has not remained constant over time; after the first wave, people probably started to consider the risk of being infected as a part of their ‘normal’ life, so they started to comply less with rules and voluntary restrictions on mobility and social interactions. This first result is, in our view, quite interesting. In fact, it shows that mobility in space is an intrinsic and structural characteristic of human nature, and therefore it can only be partially restricted for short periods. If we transpose this exercise to international mobility flows (between nations), we can clearly see how the flow-containment policies implemented by the destination countries are doomed to fail in the medium term. The comparison between the two indexes (the MRI and SI) shows that the SI is greater than the MRI on average. The highest difference between the two indicators is that recorded by Italy, a paradigmatic case in the study of the COVID pandemic. It is not the case, in fact, that the highest level of restriction out of the full sample of countries was adopted by Germany, Italy, France, and Greece. However, overall, the results show different patterns between countries without a clear model. It seems therefore that, although to a lesser extent, the mobility patterns during COVID seem to follow—after the first phase of restrictions—a sort of random process. In this context it seems that, after the end of the first wave in May 2020, the SI overestimates the level of restriction for Italy, extending the regional level of restriction to the whole national territory. We can therefore infer that the MRI works quite well for measuring and detecting the changes in the pattern of daily mobility. This aspect allows us to formulate a set of considerations regarding the usefulness of the MRI instrument and the potential of what has been observed in prospective terms. Having an index capable of capturing changes in mobility patterns and levels due to exogenous shocks can in fact lay the groundwork for simulating future mobility scenarios in which exogenous shocks may differ in terms of both the nature of the event and its intensity. What would happen, in fact, if governments decided to set policies to curb mobility due to the energy crisis or to contain ecological impacts? These results, suitably developed and modelled, can serve as a basis, and the MRI can serve as a cognitive tool. Of course, we are reasoning in the short term because in the medium and long term, we have seen how the individual’s propensity for mobility seems to take over, and this is in line with human history. The mobility of the human population has always contracted in times of war, disease, and economic crisis and then resumed vigorously. However, it is precisely in the short term that certain types of policies have to be adopted; think, for example, of the effects on mobility of exogenous shocks associated with extreme natural events. Finally, what we call fast demography, i.e. mobility, is not entirely independent of slow demography. In fact, temporary mobility and migration are closely linked and interconnected phenomena (Miller 2004). However, these phenomena can have important effects on the population and its overall dynamics by altering its propensities and behaviour significantly. Nor should the economic dimension of mobility be underestimated. In fact, this type of approach can also be extended to the mobility of goods and capital and, arguably, to trade, which is studied through approaches that are also used in the study of human mobility such as, for example, gravitational models. Moreover, we have to underline that changes in mobility patterns could imply significant changes in the structure of consumer behaviours (many people moved to the online market). The idea, therefore, is also to exploit this approach to predict the volume and direction of future trade in goods.

## Footnotes

1 Note that the Google mobility data are currently available up to 15 October 2022.
